# Supplementary material for: Factors and mediators impacting the number of undergraduate research mentees at a research-intensive Hispanic-serving institution
Source: PLoS One. 2024 Oct 31;19(10):e0289386. doi: 10.1371/journal.pone.0289386 (PMC11527152; doi:10.1371/journal.pone.0289386)
Supplement: S1 Appendix — (DOCX) [file pone.0289386.s001.docx]

**Appendix**

**Table A1. Imputation algorithm for counterfactual estimation**

| **Step 1** | Fit a polytomous GLM for number of mentored students using funding, the benefits and barriers factors and control variables using just the original (observed) data. |
| --- | --- |
| **Step 2** | Generate new data by repeating each observation in the original data five times and substituting the original observed level for funding with the remaining levels of funding. Given that there are 6 levels of funding (0, less than 20K, 20K-50K, 50K-150K, 150K-500K, and more than 500K), this means there are an additional 5 observations in the data that just have the alternative (unobserved) levels of funding. At this stage, the new data will have 5*# of rows of original data observations. |
| **Step 3** | Using the estimated model of step 1, impute the response variables using the “extra” observations produced in step 2, eg $Y\left( x,M\left( x* \right) \right)$ where *x* is the original true value of funding and *x** is the new value of funding. This is accomplished by using predictions of the model based on the “new” data points. In this case, since the outcome is ordinal (numbers of students mentored), the imputed values will be non-integer values spanning the observed levels. |
| **Step 4** | Estimate the natural effect of model reported in the paper using the expanded data set. Bootstrap simulation provides estimates of standard errors used for the significance tests and interval estimates. |

**Table A2. Univariate and Multivariate Fits of Instrumental Variable Models for Number of Undergraduate Students Mentored**

|  | **Univariate** | | | **Benefits Multivariate** | | | **Barriers Multivariate** | | |
| --- | --- | --- | --- | --- | --- | --- | --- | --- | --- |
| **Characteristic** | **IRR**^1^ | **95% CI**^1^ | **p-value** | **IRR**^1^ | **95% CI**^1^ | **p-value** | **IRR**^1^ | **95% CI**^1^ | **p-value** |
| **Funding** |  |  |  |  |  |  |  |  |  |
| 0 | — | — |  |  |  |  |  |  |  |
| less than 20K | 0.74 | 0.56, 0.97 | 0.038 | 0.67 | 0.61, 0.75 | <0.001 *^##^* | 0.67 | 0.60, 0.74 | <0.001 *^##^* |
| 20K-50K | 0.64 | 0.49, 0.82 | 0.001 | 0.62 | 0.60, 0.65 | <0.001 *^##^* | 0.62 | 0.59, 0.65 | <0.001 *^##^* |
| 50K-150K | 1.24 | 1.09, 1.42 | 0.002 | 1.23 | 1.19, 1.27 | <0.001 *^##^* | 1.23 | 1.19, 1.27 | <0.001 *^##^* |
| 150K-500K | 1.13 | 0.99, 1.28 | 0.074 | 1.03 | 0.99, 1.07 | 0.127 | 1.03 | 0.99, 1.07 | 0.153 |
| more than 500K | 1.10 | 1.0, 1.22 | 0.068 | 1.04 | 1.01, 1.07 | 0.023 | 1.04 | 1.00, 1.07 | 0.040 |
| **Race** |  |  |  |  |  |  |  |  |  |
| Hispanic | — | — |  |  |  |  |  |  |  |
| White, Non-Hispanic | 0.96 | 0.83, 1.11 | 0.6 | 0.88 | 0.85, 0.90 | <0.001 *^##^* | 0.88 | 0.86, 0.90 | <0.001 *^##^* |
| Other | 0.96 | 0.87, 1.07 | 0.5 | 0.85 | 0.83, 0.88 | <0.001 *^##^* | 0.85 | 0.83, 0.88 | <0.001 *^##^* |
| **Gender** |  |  |  |  |  |  |  |  |  |
| Female | — | — |  |  |  |  |  |  |  |
| Male | 1.17 | 1.06, 1.29 | 0.002 | 1.04 | 1.01, 1.07 | 0.006 | 1.04 | 1.01, 1.08 | 0.005 |
| Other | 1.07 | 0.85, 1.34 | 0.5 | 0.94 | 0.90, 0.98 | 0.004 | 0.94 | 0.90, 0.98 | 0.002 |
| **Faculty Rank** |  |  |  |  |  |  |  |  |  |
| Assistant Professor | — | — |  |  |  |  |  |  |  |
| Associate Professor | 1.05 | 0.92, 1.19 | 0.5 | 1.04 | 1.00, 1.07 | 0.026 | 1.04 | 1.01, 1.07 | 0.022 |
| Professor | 1.01 | 0.89, 1.15 | 0.9 | 1.03 | 0.99, 1.06 | 0.126 | 1.03 | 0.99, 1.06 | 0.100 |
| Other | 1.11 | 0.89, 1.38 | 0.3 | 1.12 | 1.07, 1.17 | <0.001 *^##^* | 1.12 | 1.07, 1.17 | <0.001 *^##^* |
| **% Research Time** | 1.00 | 1.00, 1.01 | 0.2 | 1.01 | 1.01, 1.01 | <0.001 *^##^* | 1.01 | 1.01, 1.01 | <0.001 *^##^* |
| **% Admin Time** | 1.0 | 0.98, 1.00 | 0.3 | 0.99 | 0.99, 0.99 | <0.001 *^##^* | 0.99 | 0.99, 0.99 | <0.001 *^##^* |
| **College Affiliation** |  |  |  |  |  |  |  |  |  |
| Engineering | — | — |  |  |  |  |  |  |  |
| Health Sciences | 0.84 | 0.70, 1.02 | 0.074 | 0.84 | 0.80, 0.88 | <0.001 *^##^* | 0.83 | 0.79, 0.88 | <0.001 *^##^* |
| Liberal Arts | 0.94 | 0.79, 1.13 | 0.5 | 0.95 | 0.92, 0.98 | 0.004 | 0.95 | 0.92, 0.98 | 0.003 |
| Science | 0.99 | 0.84, 1.18 | >0.9 | 0.94 | 0.91, 0.97 | <0.001 *^#^* | 0.94 | 0.90, 0.97 | <0.001 *^#^* |
| **Benefits/Barriers IV** |  |  |  | 0.97 | 0.96, 0.98 | <0.001 | 1.01 | 1.00, 1.02 | 0.002 |

*^#^* Šidàk *Corrected p-value<0.05, ^##^* Šidàk *Corrected p-value<0.01*

**Table A3. Univariate and Multivariate Causal Model Fits for Number of Graduate Students Mentored**

|  | **Univariate** | | | **Benefits Multivariate** | | | **Barriers Multivariate** | | |
| --- | --- | --- | --- | --- | --- | --- | --- | --- | --- |
| **Characteristic** | **IRR**^1^ | **95% CI**^1^ | **p-value** | **IRR**^1^ | **95% CI**^1^ | **p-value** | **IRR**^1^ | **95% CI**^1^ | **p-value** |
| **Funding** |  |  |  |  |  |  |  |  |  |
| 0 | — | — |  |  |  |  |  |  |  |
| less than 20K | 0.98 | 0.73, 1.29 | >0.9 | 0.988 | 0.91, 1.07 | 0.758 | 0.989 | 0.92, 1.07 | 0.792 |
| 20K-50K | 1.01 | 0.79, 1.29 | >0.9 | 0.984 | 0.92, 1.05 | 0.625 | 0.981 | 0.92, 1.05 | 0.566 |
| 50K-150K | 1.17 | 1.00, 1.37 | 0.050 | 1.173 | 1.12, 1.23 | <0.001 *^##^* | 1.173 | 1.12, 1.23 | <0.001 *^##^* |
| 150K-500K | 1.19 | 1.03, 1.38 | 0.023 | 1.199 | 1.14, 1.26 | <0.001 *^##^* | 1.196 | 1.14, 1.26 | <0.001 *^##^* |
| more than 500K | 1.05 | 0.93, 1.18 | 0.4 | 1.059 | 1.01, 1.11 | 0.021 | 1.056 | 1.01, 1.11 | 0.032 |
| **Race** |  |  |  |  |  |  |  |  |  |
| Hispanic | — | — |  |  |  |  |  |  |  |
| White, Non-Hispanic | 0.98 | 0.84, 1.14 | 0.8 | 0.959 | 0.91, 1.01 | 0.126 | 0.955 | 0.91, 1.01 | 0.091 |
| Other | 1.04 | 0.94, 1.16 | 0.5 | 1.023 | 0.98, 1.07 | 0.295 | 1.022 | 0.98, 1.07 | 0.306 |
| **Gender** |  |  |  |  |  |  |  |  |  |
| Female | — | — |  |  |  |  |  |  |  |
| Male | 1.05 | 0.96, 1.16 | 0.3 | 0.994 | 0.96, 1.04 | 0.783 | 0.995 | 0.95, 1.04 | 0.813 |
| Other | 0.79 | 0.60, 1.02 | 0.091 | 0.810 | 0.74, 0.89 | <0.001 *^##^* | 0.814 | 0.74, 0.90 | <0.001 *^##^* |
| **Faculty Rank** |  |  |  |  |  |  |  |  |  |
| Assistant Professor | — | — |  |  |  |  |  |  |  |
| Associate Professor | 1.02 | 0.90, 1.16 | 0.8 | 1.011 | 0.97, 1.06 | 0.633 | 1.007 | 0.96, 1.05 | 0.769 |
| Professor | 1.07 | 0.94, 1.22 | 0.3 | 1.035 | 0.99, 1.08 | 0.127 | 1.037 | 0.99, 1.09 | 0.109 |
| Other | 1.00 | 0.79, 1.25 | >0.9 | 0.982 | 0.92, 1.05 | 0.618 | 0.977 | 0.91, 1.05 | 0.532 |
| **% Research Time** | 1.00 | 1.00, 1.01 | 0.5 | 1.001 | 0.99, 1.00 | 0.630 | 1.001 | 0.99, 1.00 | 0.583 |
| **% Admin Time** | 1.01 | 1.00, 1.02 | 0.091 | 1.007 | 1.00, 1.01 | 0.001 *^#^* | 1.006 | 1.00, 1.01 | <0.001 |
| **College Affiliation** |  |  |  |  |  |  |  |  |  |
| Engineering | — | — |  |  |  |  |  |  |  |
| Health Sciences | 1.01 | 0.83, 1.23 | >0.9 | 1.039 | 0.97, 1.12 | 0.299 | 1.041 | 0.97, 1.12 | 0.278 |
| Liberal Arts | 0.97 | 0.80, 1.19 | 0.8 | 0.999 | 0.94, 1.06 | 0.966 | 1.000 | 0.94, 1.06 | 0.994 |
| Science | 1.02 | 0.86, 1.22 | 0.8 | 0.975 | 0.92, 1.04 | 0.420 | 0.980 | 0.92, 1.04 | 0.513 |
| **Benefits/Barriers IV** |  |  |  | 0.939 | 0.92, 0.96 | <0.001 *^##^* | 1.012 | 1.00, 1.02 | 0.011 |

*^#^* Šidàk *Corrected p-value<0.05, ^##^* Šidàk *Corrected p-value<0.01*
